# A comparison of stress levels, coping styles and psychological morbidity between graduate-entry and traditional undergraduate medical students during the first 2 years at a UK medical school

**DOI:** 10.1186/s13104-017-2395-1

**Published:** 2017-02-13

**Authors:** R. Zvauya, F. Oyebode, E. J. Day, C. P. Thomas, L. A. Jones

**Affiliations:** 1 Institute of Clinical Sciences, College of Medical and Dental Sciences, University of Birmingham, Birmingham, UK; 2 National Addiction Centre, King’s College London, London, UK; 3 Department of Psychological Medicine, University of Worcester, Worcester, UK; 4 Institute of Clinical Sciences, College of Medical and Dental Sciences, University of Birmingham, Birmingham, B15 2TT UK

## Abstract

**Background:**

Stress levels and psychological morbidity are high among undergraduate medical students (UGs), but there is a lack of research into the psychological health of UK graduate-entry medical students (GEs). GEs are likely to experience different (perhaps more severe) stressors and to cope with stress differently. We compared stress levels, psychological morbidity and coping styles in GE versus UG medical students studying at the same UK medical school in the same academic year.

A cross-sectional self-rated questionnaire study of all first- and second-year GE and UG medical students was conducted. Perceived stress, psychological morbidity, recent adverse life events, stress-related personality traits and coping styles were assessed using standard questionnaires.

**Results:**

75% GEs and 46% UGs responded to the questionnaire. Both groups reported equally high levels, and similar profiles of, perceived stress and psychological morbidity. Levels of recent adverse life events and stress-related personality traits were similar in both groups. Compared to UGs, GEs were more likely to use active coping (p = 0.02) and positive reframing (p = 0.03), but were also more likely to use substances (alcohol and other drugs; p < 0.001) to help them cope. Unlike UGs, second-year GEs showed less perceived stress (p = 0.007) and psychological morbidity (p = 0.006) than first-year GEs although levels of both were still high.

**Conclusion:**

Our results show that both GE students and their younger UG counterparts on a traditional medical course have similar profiles of stress symptoms. They do, however, cope with stress differently. GEs are more likely to use active problem-focused coping strategies, and they are also more likely to cope by using substances (alcohol or other drugs). GE students need interventions to prevent maladaptive coping styles and encourage adaptive coping that are tailored to their needs. Such interventions should be targeted at first-year students. It is vital that these students develop positive coping skills to benefit them during training and in a future career that is inherently stressful.

## Background

It is well-documented that medical students experience high levels of stress and psychological morbidity, such as depression [1–5]. However, there has been little work on the psychological health of graduate-entry medical students (GEs) [6]. Several UK medical schools have set up fast-track 4-year graduate entry medical degree courses in recent years [7]. Places are currently fewer than those on traditional five-year undergraduate medical courses, but given the attributes that GEs bring to medical training (such as maturity and prior learning) [8] and initial positive outcomes [9, 10], numbers may rise. It is crucial that consideration is given to the psychological health and welfare needs of this important group of students.

Graduate-entry medical students are distinct from students on traditional undergraduate medical courses (UGs) in many ways, and thus are likely to experience different stressors and manage stress in different ways. The most obvious difference is that all GEs have previously completed a first degree and are older (more mature) than UGs who tend to be school-leavers or at most have taken a ‘gap year’. Furthermore, GE programmes aim to widen participation in medical education by recruiting those who were unable to enter medical school straight after secondary education due to relatively poor A-level grades, or those who were unsure of their career choice after A-levels. Therefore they are more likely to recruit from groups that are under-represented in traditional UG medical degrees and more likely to drop out of medical training [11], namely, males and those in lower socioeconomic groups [10].

Many GEs have given up careers in other fields, which, coupled with the severe financial costs of prolonged postgraduate study [12], suggests that pressure to successfully complete the medical degree must be extremely high. Given their increased age, GEs may have other life commitments to consider such as families and financial responsibilities. Those without offspring may well be concerned about how they will combine their studies and subsequent general and specialist training with parenthood. This could mean they have less time and/or fewer resources to dedicate to their studies, which, added to the shorter duration of their medical training, could lead to high stress levels. A study of Canadian medical students on a traditional course indicated that those who were graduates experienced higher levels of stress than non-graduate students [5].

Moreover, GE courses tend to emphasise self-directed learning (SDL), often problem-based learning (PBL), which might cause particular challenges for students, particularly if their previous degree was taught didactically. GEs may well arrive at medical school with a fixed learning style that requires perseverance to change. A UK study of UG medical students on a self-directed PBL course found that stressors were related to uncertainty about individual study behaviour, progress and aptitude, assessment and availability of resources [3].

In addition to having different and perhaps more severe stressors, given their maturity and greater life experience [13], GEs are likely to use different strategies for coping with stress compared to their UG counterparts. Models of stress emphasise the importance of threat appraisal and coping in an individual’s response to stressful situations [14, 15]. Therefore, helping students to learn how to manage (cope with) stress is an important aspect of medical training. Studies have shown high levels of stress and mental illness among qualified doctors, [16, 17] which are significantly associated with similar problems at medical school [18, 19]. Hence, effective coping techniques developed at medical school will benefit students during their training and in their future professional life, and will ultimately contribute to doctors providing the best possible care for patients.

It is likely then that GEs have specific welfare needs which may be different to those of UG medical students, and that they need student support systems tailored to their requirements. A study of GEs in Australia, for example, identified that time management and finances were significant stressors and that peer support (a ‘buddy programme’, which offered academic, social and emotional support) reduced stress levels in first-years [20]. However, to the best of our knowledge, these issues have not yet been explored in new entrant GEs in the UK. Here, we compare perceived stress levels, psychological morbidity, adverse life events and coping styles in GE versus UG medical students studying at the same UK medical school in the same academic year.

## Methods

The GE course at this Medical School has been running alongside the traditional undergraduate MB ChB course since 2003. Each year, approximately 40 students are admitted to the GE course and 400 to the UG course. All GEs have at least an upper second-class (usually a first-class) honours first degree in a life science discipline. The vast majority of UGs do not have a previous degree (>95%). GEs are taught separately from UGs during the first year, and from their second year onwards they are integrated with third-year UGs. GEs are taught using PBL in small groups in the first year while UGs are taught a systems-based course by primarily lecture-based and small group traditional didactic methods. The GE course involves integrated teaching and heavily depends on SDL during the first year. The aim of the first year of the GE course is to enable GEs to enter the third-year UG programme with the same level of skills and knowledge as the UGs have after 2 years.

We conducted a cross-sectional self-rated questionnaire study during the academic year. All first- and second-year GEs (N = 85) and UGs (N = 750) were invited to participate. They were approached at the end of a compulsory lecture in the week prior to the Easter vacation. All students present were given a hard copy of the questionnaire plus detachable information pack about the study and asked to return the questionnaire to a locked box in the School Office, which was accessible 24-h a day, seven days a week including during the vacation. Students were given the opportunity to ask questions about the study before deciding whether or not to participate in the study. The data were collected anonymously. The information given to the students explained that by completing the questionnaire students were consenting to participate in the study. In order to include students who were absent from the lecture, all students received an email about the study noting that blank copies of the questionnaire pack were available for collection. All students received a reminder email in the week following the Easter vacation.

The questionnaire pack comprised the following well-validated and widely-used measures: (i) General Health Questionnaire (GHQ-12) [21] to measure psychological morbidity during the last few weeks; (ii) Perceived Stress Scale (PSS-10) [22] to measure perceived stress during the previous month; (iii) Brief COPE [23] to measure characteristic coping styles; (iv) Brief Life Events Questionnaire (BLEQ) [24] to measure recent adverse life events (last 6 months); (v) Eysenck Personality Questionnaire [25] (EPQ) to measure relevant personality dimensions, namely neuroticism and extraversion/introversion; and, (vi) general questions about sociodemographics and personal/family history of mental illness. Completion time was 20–30 min. All questionnaires were scored using standard procedures. Minor changes were made to BLEQ items to ensure relevance to university students. The items about losing a job and seeking work without success were substituted for items relating to having university examinations, and failing university examinations.

Data were analysed using SPSS v15. Categorical data were compared between GE and UG groups using Chi square tests. Due to significant deviations from the normal distribution, continuous data were compared between GEs and UGs using Mann–Whitney U tests and medians, inter-quartile ranges (IQR) and ranges are reported. Where samples were stratified into more than two groups, Kruskal–Wallis tests were used to compare continuous measures. Within groups, associations between continuous variables were examined using Spearman correlations. All tests were considered significant where p < 0.05.

## Results

### Response rates

75% (64/85) GEs and 46% (346/750) UGs responded. There was no significant difference in the sex distribution of respondents versus non-respondents in the GE cohort (χ^2^ = 0.99, p = 0.75). However, in the UG cohort, compared to non-respondents, there were significantly more female (69 versus 45%) and fewer male (31 versus 65%) respondents (χ^2^ = 28.52, p < 0.0001). There were significantly more white respondents and fewer non-white respondents compared to non-respondents in both the GE (88% respondents were white, compared to 63% non-respondents, χ^2^ = 5.54, p = 0.02) and UG groups (71% respondents were white, compared to 50% non-respondents, χ^2^ = 33.95, p < 0.0001). There was no significant difference in median age between respondents and the entire year groups in either cohort.

### Sociodemographics

Sociodemographic variables in both groups of respondents are summarised in Table 1. As expected, median age was significantly higher in GEs (24 years) compared to UGs (19 years) (U = 293.0, p < 0.001). 77% GEs were aged between 22 and 26 years and 96% UGs were aged between 18 and 21 years. Significantly more GEs than UGs were male (44 versus 31% respectively; χ^2^ = 3.83, p = 0.05). Significantly more GEs (16%) than UGs (2%) were married/cohabiting (χ^2^ = 27.84, p < 0.001). There were significant differences between the two groups in term-time living arrangements (χ^2^ = 64.99, p < 0.001). Fewer GEs (38%) than UGs (67%) lived with friends. GEs were more likely than UGs to live alone or with a spouse/partner (28 versus 7% respectively). The majority of GEs were in paid employment in the year before starting the medical degree (52%) while the majority of UGs were in full-time education (75%) (χ^2^ = 117.04, p < 0.001). There were no significant differences between groups on other sociodemographic measures: both groups were predominantly white ethnicity and predominantly Christian or no religion; and the majority of students in both groups had no reported personal history of mental illness, no reported family history of mental illness, no serious physical illness, and no children.

**Table 1:** Sociodemographics in GE and UG students

*N*	GE	UG	p
	64	346	
Year of study			
1st	38 (59.4%)	168 (48.6%)	0.11
2nd	26 (40.6%)	178 (51.4%)	
Sex			
Male	28 (43.8%)	108 (31.2%)	0.05
Female	36 (56.3%)	238 (68.8%)	
Age (yrs)			
Median	24	19	<0.001
Mode	19	25	
IQR	3	1	
Range	21–32	18–31	
Marital status			
Single	54 (84.4%)	338 (98.0%)	<0.001
Married/cohabiting	10 (15.6%)	7 (2.0%)	
Children			
No	60 (96.8%)	340 (98.6%)	0.42
Yes	3 (3.2%)	5 (1.4%)	
Ethnicity			
White	56 (87.5%)	245 (70.8%)	0.31
Other	8 (12.5%)	101 (29.2%)	
Religion			
Christian	35 (54.7%)	182 (52.6%)	0.26
None	24 (37.5%)	94 (27.2%)	
Other	5 (7.8%)	70 (20.0%)	
Living arrangements (term time)			
Alone	9 (14.1%)	23 (6.7%)	<0.001
With friends	24 (37.5%)	232 (67.2%)	
With spouse/partner	9 (14.1%)	1 (0.3%)	
With family	5 (7.8%)	51 (14.8%)	
Other	17 (26.6%)	38 (11.0%)	
Psychiatric illness			
No	57 (90.5%)	326 (94.8%)	0.15
Yes	6 (9.5%)	18 (5.2%)	
Family history of psychiatric illness			
No	47 (73.4%)	223 (64.8%)	0.38
Yes	14 (21.9%)	94 (27.3%)	
Unsure	3 (4.7%)	27 (7.8%)	
Serious physical illness			
No	64 (100%)	337 (97.4%)	0.21
Yes	0	9 (2.6%)	
Year before starting medical degree			
Studying FT	23 (35.9%)	261 (75.4%)	<0.001
Gap year	5 (7.8%)	61 (17.6%)	
FT employment	33 (51.6%)	21 (6.0%)	
Other	3 (4.7%)	3 (0.9%)	

### GHQ-12

**Fig. 1:**
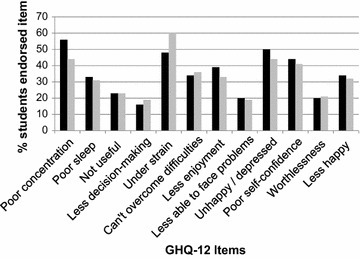
Frequency of GHQ-12 items endorsed by graduate-entry course (GEC) and undergraduate (UG) course medical students. GEC (*filled square*), UG (*open square*)

There was no significant difference between the GHQ-12 scores (scored using the Likert method, maximum score 36) of the GEs and UGs (U = 10744.5, p = 0.85; Table 2). GHQ-12 ‘caseness’ (indicative of probable psychiatric disorder) was scored using the GHQ scoring method. A cut-off point of 3–4 was used for comparison with other studies of UK medical students [3, 12]). 52% (N = 33) GEs and 46% (N = 159) UGs scored above the caseness threshold (χ^2^ = 0.59, p = 0.44). We explored this further by comparing groups on each GHQ-12 item, but no significant differences were found. In both groups the most frequently endorsed items (Fig. 1) were: being able to concentrate less than usual (56% GE and 44% UG); feeling constantly under strain (48% GE and 60% UG); and feeling more unhappy or depressed than usual (50% GE and 44% UG). In both groups more females (25/33, 76% GE and 122/159, 77% UG) than males were GHQ-12 cases and there was no significant difference in this distribution between groups (χ^2^ = 0.03, p = 0.86). There was no significant difference in median age between GHQ-12 cases and non-cases in either group.

**Table 2:** GHQ-12, PSS-10, BLEQ and EPQ scores in GE and UG students

***N***	GE	UG	p
	64	346	
General Health Questionnaire (GHQ-12)			
Median	14	14	0.85
IQR	9	9	
Range	5–35	2–34	
Perceived Stress Scale (PSS-10)			
Median	14	17	0.13
IQR	11	10	
Range	2–38	1–38	
Brief Life Events Questionnaire (BLEQ)			
Median	4	5	0.17
IQR	5	4	
Range	1–16	0–25	
EPQ—Extraversion			
Median	14	14	0.43
IQR	7	7	
Range	3–21	2–21	
EPQ—Neuroticism			
Median	11	12.5	0.20
IQR	12	8	
Range	1–23	0–23

### PSS-10

There was no significant difference between the PSS-10 scores of GEs and UGs (U = 9169.0, p = 0.13; Table 2). The most frequently endorsed items in both groups were: feeling nervous and ‘stressed’ (44% GEs and 51% UGs reported ‘often’ feeling like this); and feeling unable to cope with things (33% GEs and 30% UGs reported ‘often’ feeling like this). In both groups, females had significantly higher PSS-10 scores than males. There were no significant differences between groups when we stratified by sex. There was no significant correlation between age and PSS-10 score in either group.

### BLEQ

The number and severity of life events, as measured by BLEQ, did not differ significantly between the two groups (U = 7189.5, p = 0.17; Table 2). We examined each life event separately to see if there were different profiles of life events in GEs compared to UGs, and one significant difference emerged. On the most frequently endorsed item, regarding impending university examinations, fewer GEs reported they were severely affected by this event (29% GE versus 45% UG; χ^2^ = 6.21, p = 0.045). In both groups, females had significantly higher BLEQ scores than males. There were no significant differences between groups when we stratified by sex. There was no significant correlation between age and BLEQ score in either group.

### EPQ

There were no significant differences between the scores of GEs and UGs on the extraversion (U = 9584.0, p = 0.43) and neuroticism (U = 9547.5, p = 0.20) dimensions of the EPQ (Table 2). In both groups, females had significantly higher neuroticism scores than males. There were no significant differences between groups when we stratified by sex. There was no significant correlation between age and neuroticism score and age and extraversion score in either group.

### Brief COPE

There were significant differences in the coping strategies used by the two cohorts of students (Table 3). GEs were less likely to use religion as a coping mechanism than UGs (U = 9044.5, p = 0.01). This significant difference held when we compared only those students who reported their religion as Christian (U = 2519.5, p = 0.04). Sample sizes were not large enough to allow us to stratify groups by other religions. GEs were more likely than UGs to use active coping (U = 8964.5, p = 0.02), substances (U = 8122.5, p < 0.0001) and positive reframing (U = 9180.0, p = 0.03). There were no significant differences between the groups in the use of self-distraction, denial, emotional support, instrumental support, behavioural disengagement, venting, planning, humour, acceptance and self-blame.

**Table 3:** Brief COPE scores in GE and UG students

***N***	GE	UG	p
	64	346	
Self-distraction			
Median	5	5	0.06
IQR	2	1	
Range	2–8	2–8	
Active coping			
Median	6	5	0.02
IQR	3	2	
Range	3–8	2–8	
Denial			
Median	2	2	0.20
IQR	0	1	
Range	2–5	2–8	
Substance use			
Median	3.5	2	<0.001
IQR	2	2	
Range	2–8	2–8	
Emotional support			
Median	5	5	0.69
IQR	3	3	
Range	2–8	2–8	
Instrumental support			
Median	5	5	0.85
IQR	2	3	
Range	2–8	2–8	
Behavioural disengagement			
Median	2	2	0.76
IQR	1	1	
Range	2–6	2–8	
Venting			
Median	4	4	0.87
IQR	3	2	
Range	2–8	2–8	
Positive reframing			
Median	6	5	0.03
IQR	2	2	
Range	3–8	2–8	
Planning			
Median	6	6	0.052
IQR	2	2	
Range	3–8	2–8	
Humour			
Median	4	4	0.57
IQR	2	3	
Range	2–8	2–8	
Acceptance			
Median	6	6	0.76
IQR	2	2	
Range	3–8	2–8	
Religion			
Median	2	2	0.01
IQR	1	3	
Range	2–8	2–8	
Self-blame			
Median	5	5	0.74
IQR	2	2	
Range	2–8	2–8	

**Fig. 2:**
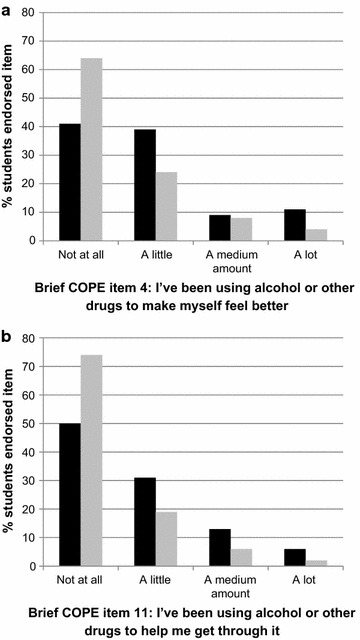
Frequency of Brief COPE responses to item 4 (Graph A) and item 11 (Graph B) by graduate-entry course (GEC) and undergraduate (UG) course students. GEC (*filled square*), UG (*open square*)

We examined the items related to substance use in more detail (Fig. 2). On both items GEs were significantly more likely than UGs to use alcohol or drugs as a coping strategy (Item 4 χ^2^ = 14.97, p = 0.002; Item 11 χ^2^ = 15.51, p = 0.001). Fewer than half GEs (N = 26, 41%) reported ‘never’ using alcohol or drugs to make themselves feel better, compared to around two-thirds UGs (N = 220, 64%). Seven (11%) GEs reported doing this ‘a lot’, compared to 13 (4%) UGs. Half GEs (N = 32, 50%) reported using alcohol or drugs at least a little to help them get through it, compared to a quarter UGs (N = 91, 26%). Four (6%) GEs reported using this coping strategy ‘a lot’, compared to 8 (2%) UGs. We repeated these analyses including only those students who reported their religion as ‘Christian’ or ‘no religion’ and found exactly the same pattern of significant results.

We found some significant differences in coping when we stratified by sex. Male GEs were significantly more likely than their male UG counterparts to use active coping (U = 1092.0, p = 0.03), substances (U = 979.5, p = 0.001) and planning (U = 1023.0, p = 0.007). Female GEs were significantly more likely than female UGs to use substances (U = 3418.0, p = 0.03) and were significantly less likely to use religion (U = 3292.5, p = 0.01). Within the GE cohort, the only significant difference in coping was that males were significantly more likely than females to use planning (U = 305.5, p = 0.01). There were no significant sex differences in coping within the UG group.

There were no significant correlations between age and any of the coping scores in the UG group, however within the GE cohort age was significantly positively correlated with acceptance (rs = 0.28, p = 0.03) and significantly negatively correlated with denial (rs = −0.29, p = 0.02), emotional support (rs = −0.31, p = 0.02), and venting (rs = −0.31, p = 0.02). The correlation between age and substance use was −0.14 (p = 0.28).

We examined correlations between the coping scores and GHQ-12 scores within the GE group. We found significant moderate positive correlations between GHQ-12 score and self-distraction (rs = 0.28, p = 0.03), denial (rs = 0.37, p = 0.02), substance use (rs = 0.40, p = 0.001), behavioural disengagement (rs = 0.54, p < 0.001) and self-blame (rs = 0.45, p < 0.001). There was a significant moderate negative correlation between GHQ-12 score and active coping (rs = −0.26, p = 0.04).

### First- versus second-years

Comparing GE and UG first-year students only, there were no significant differences between GHQ-12 scores, PSS-10 scores, BLEQ scores or GHQ-12 caseness (66 versus 50% respectively). First-year GEs were significantly more likely to use active coping (p = 0.04) and substances to cope (p < 0.0001) than first-year UGs.

We compared stress levels and coping within each cohort (GE and UG) stratified by year of the course (year 1 versus year 2). We found no significant differences on any of the measures between first- and second-year UGs. However, there were significant differences in the GE cohort. Compared to second-years, first-years had significantly higher GHQ-12 scores (medians were16 (IQR = 9) versus 10 (IQR = 7) respectively, U = 258.0, p = 0.001) and PSS-10 scores (medians were 18 (IQR = 12) versus 11 (IQR = 9) respectively, U = 265.5, p = 0.007). 66% (N = 25/38) first-year GEs and 31% (N = 8/26) second-years met the criteria for GHQ-12 ‘caseness’ (χ^2^ = 7.58, p = 0.006). There were no significant differences on any of coping scales.

## Discussion

This is the first comparison of stress, coping and psychological morbidity between GE students and traditional undergraduate medical students at a UK medical school. As previous studies have shown, [3, 12, 26] we found high levels of stress and psychological morbidity among our students, but these were equally high in GEs compared to UGs. In line with previous studies, we found higher levels of stress and morbidity among female students compared to males but this was the same in both course groups. We did not find a different profile of stress in GEs. Like UGs, poor concentration, feeling under strain and feeling unhappy were particular problems. A significant number of students in both groups felt nervous and ‘stressed’ and perceived difficulty coping. Likewise, levels of stress-related personality traits were similar in both groups. Contrary to expectation, there was not a greater number, or different profile, of recent adverse life events among GEs. The most frequent life event endorsed in both groups was university examinations. Interestingly, fewer GEs felt severely affected by the examinations, which may be due to previously learned coping strategies.

The strategies used to cope with stress differed, with GEs more likely to use ‘positive’ or ‘problem-focused’ approaches of active coping (i.e., taking action to try to make a stressful situation better) and positive reframing (i.e., reappraising a stressful situation to see it in a positive light) than UGs. UGs were more likely to use religion (i.e., praying or meditating) as a way of coping and this was not due to the greater number of students following non-Christian religions in this group. While it is reassuring that among the most common coping strategies in both groups were problem-focused approaches, i.e., active coping and planning (i.e., thinking about what steps to take to improve a stressful situation), it was alarming that significantly more GEs used substances to help them cope (around 50–60%, compared to around one-third of UGs). Again, we did not find that the increased use of substances to cope was due to greater representation of religions that might forbid the use of alcohol and other drugs among UGs, and the increased use among GEs was significant for both male and female students.

The high rates of possible psychological morbidity (52% GE and 46% UG) in our students is worrying, but not dissimilar to rates in other studies of medical students [2, 3, 12]. For example, in Manchester around one-third of students were GHQ cases, [26] (Guthrie et al.) and in Glasgow 52% first-year medical students were GHQ cases by the third term [3]. By way of comparison, the 2003 Health Survey for England found 10–12% GHQ-12 caseness (using 3–4 cut-off as we have done here) in men and 15–16% in women in the same age-groups as our students [27]. Our primary focus was not on exploring reasons for the high levels of psychological morbidity and perceived stress. Other authors have explored these in UG medical students and important contributory factors have been shown to be course-related, [3, 4, 28] financial, [12] and personal, such as, having a lack of social support or stress-related personality traits [1, 29, 30]. Similarly, we did not explore reasons for the lower stress levels and lower (although still high at 31%) rate of psychological morbidity in second-year GEs. We suggest that this may be due to a ‘settling down’ of the stress associated with starting the medical course (a major life event) or a consequence of starting clinical training (second-year GEs join the third-year of the UG MB ChB course). A previous study at St George’s Hospital Medical School showed that third-year GEs were less anxious and more prepared for the transition to clinical years than UGs [31]. We did include measures of known stress-related personality traits (e.g., neuroticism) and a measure of recent adverse life events but we found no differences between GEs and UGs, and no differences between first- and second-year GEs. Therefore, personality and known stressors (such as bereavement, relationship problems, and serious illness in self or family) do not account for the differences observed here. It was interesting that we did not find as high a prevalence as expected of potential stressors in the GE group, for example, 3% had children, 16% were married/co-habiting and around half were in full-time employment prior to starting the course. Future longitudinal work is required to explore stressors in GEs in more detail.

Of greater interest here is the increased use of substances among GEs, both males and females, to ‘make them feel better’ and to ‘help them get through’. Other reports have demonstrated high use of alcohol, cannabis and other illegal drugs among UG medical students in the UK [32, 33]. Understanding students’ coping strategies is at least as important as understanding the stressors they face. Stress during medical training, as in life in general, is inevitable and can have positive consequences, such as increasing motivation. It is only when stress is too great or unresolved (by poor coping) that it leads to negative effects on cognitive functioning, such as concentration and decision-making, and on emotional and physical health [34]. Indeed in our dataset, although we cannot infer cause and effect from these cross-sectional associations, there were relationships between increased psychological morbidity score and dysfunctional coping strategies (such as, using substances, denial and disengagement) and an inverse relationship between psychological morbidity score and active coping. It is important that effective interventions tailored to specific groups of students are developed to help students cultivate adaptive coping strategies and to promote good mental health. Indeed, an impetus for introducing medical training for graduates was the hope that their motivation would be higher and the attrition rate would be lower [35], so it is essential that we are concerned with the psychological health of our students to ensure this aspiration is realised.

Our data suggest that the first-year of study for GEs is a time of particular risk and interventions should target this period. Interventions might be psychoeducational literature about effective coping or expert-led workshops. The greater use of active psychological strategies, such as positive reframing, among GEs suggests that interventions based on cognitive-behavioural therapeutic (CBT) principles may be successful in this group. We do not know whether the coping characteristics shown by GEs in this study are simply due to their increased age, but to partial out the effect of age in the analysis would be throwing the baby out with the bathwater given that increased (chronological) maturity is an important defining feature of GEs. We did test the correlations between coping strategies and age and found no significant association with substance use in either student group. Our analysis suggests that there are no striking differences in coping styles between male and female GE students, so any intervention could be used with the whole cohort. Various interventions for stress management at some medical schools have been implemented, but their appropriateness and effectiveness is not known [36]. It is vital that interventions are evidence-based and tailored to the needs of particular groups of students and that their effectiveness is scientifically tested.

Doctors have higher rates of mental disorder than the general population, including higher suicide rates [37]. Around 20% doctors report using substances to help them cope [38] and up to 7% will have a substance misuse problem during their lifetime [39]. It is therefore vital that good mental health is promoted early in medical schools to prepare students for a future career which is inherently stressful [40–42], to mitigate future burn-out and mental ill-health. Increasing the profile of mental health teaching, including substance misuse, [43] on medical curricula may help to highlight important issues to students and encourage them to consider their own mental health and behaviour.

Even if medical schools are unable, or unwilling, to invest in specific interventions to support and improve the psychological health of their students, our data suggest that we must ensure that existing welfare systems are supportive and open towards students with psychological problems, and that they are not seen as weak or ill-disciplined. At this medical school, students have access to personal mentors, senior welfare tutors and the University Counselling and Guidance Service, but clearly problems remain. We need students who are using substances to cope to come forward for help without fear of the consequences. If students come forward at an early stage, they can be directed to appropriate professional help before a serious problem develops. It has been shown that there are a number of barriers to help-seeking for psychological problems in medical school [1, 44]. Students feel that declaring a psychological problem is shameful and may be inviting disciplinary action and a threat to their future career (fitness to practise procedures). Confidentiality is a particular concern. Furthermore, qualified doctors with mental health disorders feel stigmatised and may have difficulty accessing appropriate healthcare [45], thus this problem needs tackling at medical school to ensure that precedents are not set and that students are well-prepared for their future career. It is very likely that there are more students coping with stress by using substances than our data suggest. Even though the questionnaires were completed anonymously, students may well have been concerned about being ‘found out’ and responded defensively as a consequence. Perhaps this was more prevalent in the younger (less mature) UGs, which could account for the significantly increased use of substances for coping found in the GE group. It would be interesting to explore the use of substances further, incorporating measures of quantity/frequency with more detailed measures of reasons for use [46], however defensive responding may prove impossible to avoid in these student groups.

Our data should be interpreted in light of a number of limitations other than those already highlighted. Although the response rate was acceptable in the GE group, the rate in the UG group was below 50%. This was despite our best efforts to engage the whole first- and second-year cohort. There was an over-representation of females among UGs who responded. Females reported higher levels of stress and psychological morbidity overall, so if more male UGs had responded we may have found that average levels in the UG group were reduced and maybe even to levels significantly lower than those of the GEs. We also had an under-representation of non-white students among our respondents in both groups. It is difficult to know how this bias affected our results and is a target for future research. We used self-report questionnaires, which although widely-used and validated, have obvious disadvantages, such as a limited pre-determined range of variables with forced-choice response options. A richer dataset could be obtained using semi-structured interviews about stress and coping. We examined our students at a single point in time, chosen to be not close to examinations for either group. However, stress levels and coping strategies are dynamic and context-dependent so a longitudinal investigation is needed. Our study was limited to one medical school in the UK, and our findings need investigation at other medical schools. Our results were not corrected for multiple testing as we did not want to miss significant effects in this exploratory study. Although the difference in the proportion of students in the GE and UG groups reporting using alcohol/drugs as a coping strategy would remain significant after correction for multiple testing, it is likely that some p values would not stand up to stringent statistical correction, therefore our findings must be viewed as preliminary and require replication.

## Conclusion

Our results show that GE students at this medical school do not experience more stress than their younger UG counterparts on a traditional medical course, and profiles of stress symptoms are similar in both groups. They do, however, cope with stress differently. GEs are more likely to use active problem-focused coping strategies, but they are also more likely to cope by using substances (alcohol or other drugs). Tailored interventions to prevent or alter maladaptive coping and teach or encourage adaptive coping styles that can be drawn upon in future professional life as a doctor are needed and should be targeted at first-year GEs. This is particularly important as the number of GE students continues to increase. Further research should involve longitudinal studies of stress and coping using qualitative interviews and more detailed exploration of substance use in GEs, and should be widened to other UK medical schools.

## Abbreviations

UGs: undergraduate medical students
GEs: graduate-entry medical students
SDL: self-directed learning
PBL: problem-based learning
GHQ-12: General Health Questionnaire
PSS-10: Perceived Stress Scale
BLEQ: Brief Life Events Questionnaire
EPQ: Eysenck Personality Questionnaire
IQR: inter-quartile range
CBT: cognitive-behavioural therapeutic
